# Dermatoglyphic meta-analysis indicates early epigenetic outcomes & possible implications on genomic zygosity in type-2 diabetes

**DOI:** 10.12688/f1000research.6923.1

**Published:** 2015-08-24

**Authors:** Seile Yohannes

**Affiliations:** 1 Department of Biology, College of Natural & Computational Sciences, Jigjiga University, Jigjiga, Ethiopia

**Keywords:** dermatoglyphics, diabetes, epigenetics, prenatal development, heterozygosity, developmental homeostasis, genetic predisposition

## Abstract

Background: Dermatoglyphic studies, particularly those arising from the Dutch Hunger Winter Families Cohort, indicate an involvement of prenatal epigenetic insults in type-2 diabetes. However, the exact orchestration of this association is not fully understood. Herein is described a meta-analysis performed based on a belief that such an approach could shed some light as to the role of genetic & epigenetic influences in the etiology of type-2 diabetes.

Methodology/principal findings: The study incorporated reports identified from PubMed, Medline, & Google Scholar databases for eligible case-control studies that assessed dermatoglyphics in type-2 diabetes cases relative to controls. Over 44,000 fingerprints & 2300 palm prints from around 4400 individuals were included in the analysis. Decreased loops patterns [OR= 0.76; 95% CI= (0.59, 0.98)], increased non-loop patterns [OR= 1.31; 95% CI= (1.02, 1.68)], and reduced absolute finger ridge counts [OR= -0.19; 95% CI= (-0.33, -0.04)] were significant findings among the diabetic group. These results are indicative of mild developmental deviances, with epigenetic insults significantly linked to early gestation wherein critical events &signaling pathways of the endocrine pancreas development are witnessed. Further, the increased loop patterns with decreased non-loop patterns were deemed as possible indicators of decreased genomic heterozygosity with concurrently increased homozygosity in the diabetic group, linked to reduced buffering capacities during prenatal development.

Conclusions: Epigenetic insults primarily during the 1 ^st^ trimester, to a lesser extent between the early-to-mid 2 ^nd^trimester, but least likely linked to those beyond the mid-second trimester are evident in type-2 diabetes. It is recommended that future research aimed at expounding the prenatal origins of T2DM, as well as developing novel therapeutic methods, should focus on the early stages of endocrine pancreatic development.

## Introduction

The dawn of the 21 ^st^ century has witnessed a trend of epidemiologic shift from acute infections to chronic degenerative disorders of intricate etiologies markedly obscure to comprehend. One such multifaceted forerunner deemed as a major global public health issue is diabetes mellitus, recording an estimated global prevalence of 8.3% in 2013 that is projected to rise to an alarming figure of over 590 million afflicted individuals by 2035 ^1^. Type-2 diabetes mellitus (T2DM) accounts for greater than 90% of all diabetics ^2^. It is a polygenic multi-factorial disorder entailing multiple endogenous & exogenous risk factors ^3, 4^.

The estimated heritability of T2DM underscores the contribution of non-genetic components in its etiologic complexity ^5^. Several prenatal environmental agents have been hypothesized as putative risk factors for T2DM, including: maternal dietary patterns, objective hardship or stress related to natural disasters, season of birth (related to vitamin D or melatonin metabolisms), urbanization, medications such as corticosteroids during pregnancy, use of pesticides, and so on ^6– 10^.

In line with this, the Dutch Hunger Winter Families Cohort studies investigate the concept of the *thrifty phenotype hypothesis*, linking gestational hunger to the offspring’s adulthood insulin resistance, glucose intolerance, and subsequent development of diabetes ^11, 12^. Such outcomes have in turn been shown to be associated with epigenetic alterations, including changes in the normal pattern of DNA methylation among genes essential for growth & development ^4, 13– 15^. Yet, further studies from varied populations originating from varied environmental settings are necessitated in order to comprehend such etiologic complexity.

A currently emerging area of study with multifarious findings of peculiar phenotypes among a broad array of chronic disorders including T2DM is dermatoglyphics (DG). It refers to the systematic study of the friction ridge features on the palmar & plantar surfaces, which are formed as complex polygenic multi-factorial traits ^16, 17^.

Development of DG begins during the 6 ^th^ week of gestation, and their basic structure remains unaffected by subsequent environmental insults following full establishment during the 24 ^th^ week ^18^. Owing to this & several other advantageous features of DG, prospects of serving as markers to explore the nature & timing of intra-uterine irregularities have been assessed ^19^. Developmental noise associated with chromosomal/gene abnormalities, environmental pressures, or a combination of these, have been shown to be detectable via DG ^20^.

Despite their pervasiveness, studies exploring DG in T2DM that entail strong deductions fortified by models that elucidate the underlying developmental mechanism in play thereby vindicating such associations, are distinctively vague. The manifold DG variable types, methodological discrepancies, as well as poorly designed or implemented studies have been deemed citable attributes to the prevalent contradicting findings ^21^.

In contrast, a momentous cluster of researchers stand-out from among this crowd, which view peculiar DG as ripple-effects of early prenatal insults behind the structural & functional defects intrinsic of T2DM *per se*, including pancreatic endocrine system (β-cell functioning) alterations ^22– 26^. Though the core of such studies contends that DG are indicative of possible genetic predispositions or epigenetic prenatal outcomes, the exact orchestration of this association is not fully understood. This impedes the strides in the etiological understanding of T2DM, rendering the concept of employability of DG in genetic epidemiology to still be subject to further debate & research.

Herein is described an exhaustive meta-analysis based on a hypothesis that an advanced statistical approach would lead to better conclusive results that will shed some light as to the role of genetic & epigenetic influences in the etiology of T2DM.

## Methods

The protocols for this work adhered to the principles of the Cochrane’s guidelines for systematic reviews ^27^, as well as the preferred reporting items for systematic reviews and meta-analyses ‘PRISMA’ ^28^. The PRISMA checklist of the study is depicted in Supplementary file S1.

### Criteria for considering studies for this review

A literature search of studies published up to July 2015 which report the association between diabetes and dermatoglyphics was performed, wherein PubMed, PubMed Central, Medline, & Google Scholar databases were exhaustively searched using synonyms and combinations of the terms, as summarized in Table 1. As a secondary option, reference lists of initially identified studies were hand-searched for putative studies, which were, once identified, looked up in the archives of journals they were published in. The data search was performed three times to ensure exhaustiveness, twice before the analysis, and a final cross-checking search after the analysis was completed based on the studies identified from the two initial searches.

The eligibility of each of the studies strictly adhered to the following criteria: (1) the article reports on an original peer-reviewed analysis; (2) it is available as a full text article published in English; (3) the article includes relevant raw data for pair-wise comparisons, mainly odds ratios (OR), confidence intervals (CI), mean values, and standard deviations (SD), or at least reported sufficient data that can be employed to estimate these parameters, including count or frequency data, accurate percentage frequencies, or standard errors of mean values.

For studies with some missing raw data or manifesting some inconsistencies hindering accurate extraction of data for meta-analysis, an attempt was made to contact the authors, which was done twice for each corresponding author within a 30 day interval via email addresses available on their articles. Data from responsive authors within the 30 days from the second contact (60 days in total) were included in the analysis.

### Dermatoglyphic measures

The continuous dermatoglyphic variables included by a recent dermatoglyphic meta-analysis on schizophrenia were adopted ^29^, and included herein in addition to a few more relevant continuous variables, as well as the inclusion of dichotomous type variables (pattern type distribution).

Thereby, three major classes of variables, each with respective subclasses, were included: Fingerprint patterns: based on the three pattern classification of fingerprint patterns as: arch, loop, and whorl patterns. Ridge counts: a count of ridges between a triradius and a core (in fingerprint patterns), or between two triradii (for palmar ridge counts). Thus the total finger ridge count (TFRC), absolute finger ridge count (AFRC), and total palmar a-b ridge count (TABRC) were assessed. Palmar Angles: angles formed by joining distal palmar inter-digital triradii a/d with the proximal palmar axial triradii t, yielding the ATD, DAT, and ADT angles ^30, 31^.

### Data extraction & summary

Qualitative and quantitative DG variables reported by each study were extracted carefully and summarized in coded spreadsheets. Pattern frequencies or means and SDs were pooled from left- & right-hand data, as well as from male and female data for each study as per previous reports ^29^. Whenever necessary, percentage frequencies were converted to counts, and SDs calculated from reported standard error of means by multiplying standard errors of means from within a treatment group by the square root of the sample size ^27^.

### Statistical analysis

Cochrane RevMan 5.3 ^32^ & MetaXL 2.2 software were used for analyses. Hedge’s g effect sizes for each continuous variable & ORs for each dichotomous variable, together with respective 95% confidence intervals, z-scores, & p-values were estimated ^27^.

Heterogeneity across studies was assessed following the I ^2^ statistics ^33^. The pooled effect size estimations were done using the fixed-effect model if heterogeneity was non-significant or the random-effects model if significant heterogeneity was shown to exist ^34– 36^.

Publication bias was investigated by visual inspections of funnel plots. Finally, sensitivity analyses for each class were performed to evaluate the influences of selected study and participant characteristics on study results.

## Results

**Figure 1.**
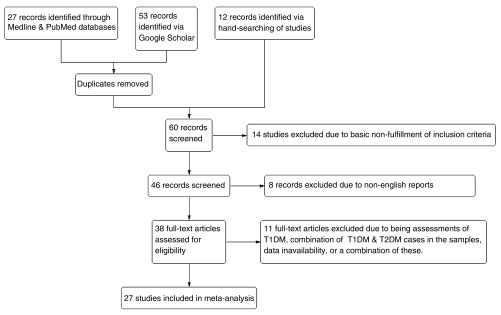
Flow diagram of search, evaluation, & exclusion/selection procedures.

The literature search ( Figure 1), which was to the best of my knowledge, exhaustive and comprehensive, yielded a total of 27 eligible studies after excluding unavailable articles, non-open-access reports, articles not in English, reviews and/or short reports, as well as those with unavailable or insufficient data. Of the 27 studies, respectively 20, 9, 4, 5, 10, 4 and 4 studies reported valid & usable data on the TFRC, AFRC, A-B RC, ATD, DAT, & ADT variables, which were incorporated into the meta-analysis. The characteristics of the 27 eligible studies ^37– 63^ are included in Table 2, while the excluded studies are elaborated in Table S1 within the Supplementary file S2.

**Table 1.** Search term combinations used to identify putative studies reporting dermatoglyphics in T2DM.

Search Term 1	Search Term 2
“Diabetes” Or ‘insulin resistance’	“Dermatoglyphics” Or “Friction ridges”
	‘fingerprint’ Or “Arch, Loop, Whorl”
	“palm print”
	“atd”
	‘ridge count’ Or “TFRC, AFRC, ARC, a-b, a-d, b-c, c-d”

**Table 2.** Characteristics of the studies included.

Author(s)	Year	Country of Study	Study Pop.	
			T2DM ^a^	Healthy
Bala *et al.* ^37^	2015	Gangtok, India	70	70
Burute *et al.* ^38^	2013	Pune, India	101	100
Desai & Hadimani ^39^	2013	Bijapur, India	100	100
Fuller ^40^	1973	London	68	825
Gabriel & Babajide ^41^	2004	Nigeria	49	52
Karim & Saleem ^42^	2014	Kurdistan, Iraq	50	50
Marera *et al.* ^43^	2015	West Uganda	150	150
Mehta & Mehta ^44^	2015	Hyderabad, India	100	100
Mittal & Lala ^45^	2013	India	100	100
Nayak *et al.* ^46^	2015	Maharashtra, India	50	50
Ojha & Gupta ^47^	2014	Rajasthan, India	100	100
Pathan & Gosavi ^48^	2011	Sholapur, India	100	100
Pathan & Hashmi ^49^	2013	Sholapur, India	100	100
Rajanigandha *et al.* ^50^	2006	India	112	142
Rakate & Zambare ^51^	2014	Maharashtra, India	350	350
Rakate & Zambare ^52^	2013	Maharashtra, India	75	75
Ravindranath & Thomas ^53^	1995	Bangalore, India	150	120
Sachdev ^54^	2012	Rajasthan, India	100	150
Sengupta & Boruah ^55^	1996	Assam, India	88	80
Sharma *et al.* ^56^	2012	Rajasthan, India	50	50
Srivastava & Rajasekar ^57^	2014	India	74	74
Sudagar *et al.* ^58^	2014	Tamilnadu, India	150	150
Sumathi & Desai ^59^	2007	Bijapur, India	100	100
Taiwo & Adebanjo ^60^	2012	Lagos, Nigeria	84	71
Trivedi *et al.* ^61^	2014	Gujarat, India	50	50
Udoaka & Lawyer-Egbe ^62^	2002	Port Haricourt, Nigeria	90	90
Umana *et al.* ^63^	2013	Zaria, Nigeria	101	126

Authorship, year of publication, country of origin of the sampled population, and sample sizes of the diabetic & healthy groups within the sampled population.

^a^Type-2 diabetes mellitus.

### Fingerprint patterns

**Figure 2.**
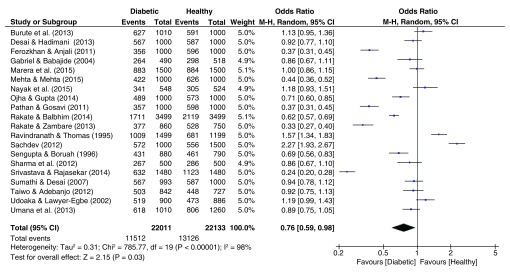
Forest plot of the association between loop fingerprint patterns & T2DM.

The pooled effect sizes for all three fingerprint pattern datasets showed that significant heterogeneity was prominent ( Figure 2– Figure 4). The OR datasets ranged from 0.24 to 2.27, 0.34 to 3.35, and 0.55 to 5.99 for the loops, whorls, and arches respectively. The pooled OR of the loops resulted in a significant effect size (OR = 0.76, 95% C.I. = 0.59–0.98, z = 2.15, p = 0.03), with decreased occurrence of loops among diabetic patients. In contrast, the pooled estimates for the whorls & arches resulted in non-significant effect sizes, with an OR of 1.30 (95% C.I. = 0.98–1.72, z = 1.83, p = 0.07) for whorls, and an OR of 1.19 (95% C.I. = 0.98–1.72, z = 1.12, p = 0.26) for the arches, indicating higher prevalence of both patterns among diabetic patients.

**Figure 3.**
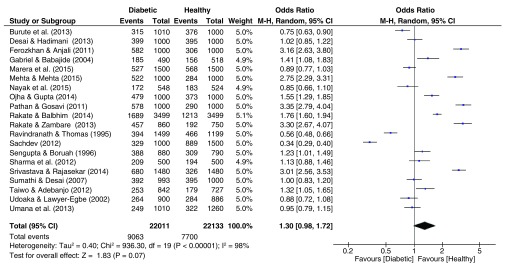
Forest plot of the association between whorl fingerprint patterns & T2DM.

**Figure 4.**
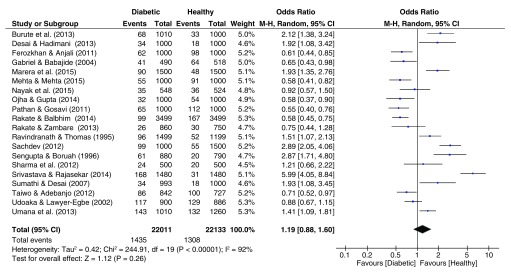
Forest plot of the association between arch fingerprint patterns & T2DM.

### Total Finger Ridge Count (TFRC)

**Figure 5.**
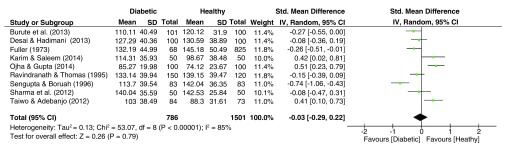
Forest plot of the association between total finger ridge count (TFRC) & T2DM.

As is evident from the forest plot of TFRC effect sizes ( Figure 5), the pooled effect sizes for the TFRC dataset were significantly heterogeneous (I ^2^ = 85%, p<.001). The pooled estimate resulted in an insignificant effect size (g = -0.03, 95% C.I. = −0.29–0.22, p = 0.79), with slightly lower TFRC among diabetic patients.

### Absolute Finger Ridge Count (AFRC)

**Figure 6.**
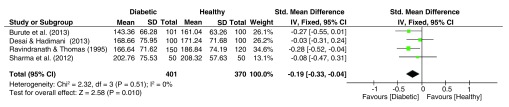
Forest plot of the association between absolute finger ridge count (AFRC) & T2DM.

The forest plot of AFRC effect sizes ( Figure 6) reveals no heterogeneity within the dataset, with the pooled estimate yielding a significant effect size (g = 0.19, 95% C.I. = -0.33–-0.04, z = 2.58, p = 0.010), with lower means prevalent among diabetic patients.

### Total A-B Ridge Count (TABRC)

**Figure 7.**
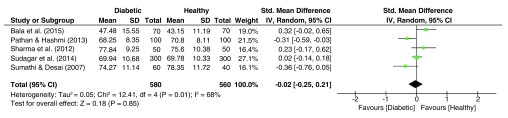
Forest plot of the association between total A-B ridge count (TABRC) & T2DM.

The pooled effect sizes for the palmar TABRC dataset ( Figure 7) was significantly heterogeneous (I ^2^ = 68%, p = 0.01), with the pooled estimate yielding slightly decreased but insignificant means among the diabetic subjects (g = -0.02, 95% C.I. = -0.25–-0.21, z = 0.18, p = 0.85).

### ATD Angle

**Figure 8.**
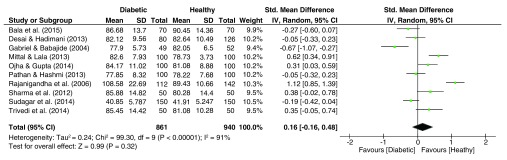
Forest plot of the association between palmar angle ATD & T2DM.

As is evident from the forest plot of TFRC effect sizes ( Figure 8), pooled effect sizes for the palmar ATD angle dataset was significantly heterogeneous (I ^2^ = 91%, p<.001). The pooled estimate resulted in a significant effect size (g = 0.16, 95% C.I. = -0.16–0.48, z = 0.99, p = 0.32), with higher ATD angles among diabetics.

### TAD Angle

**Figure 9.**
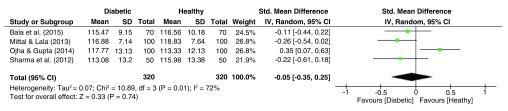
Forest plot of the association between palmar angle DAT & T2DM.

As depicted in Figure 9, significant heterogeneity was observed for the DAT angle dataset (I ^2^ = 72%, p = 0.01), with the pooled estimate showing an insignificant effect size (g = -0.05, 95% C.I. = -0.35–0.25, z = 0.33, p = 0.74), with lower values expected among the group of diabetics.

### ADT Angle

**Figure 10.**
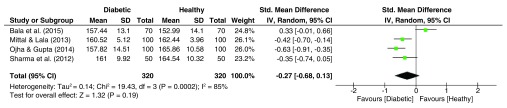
Forest plot of the association between palmar angle ADT & T2DM.

Similar to the DAT angle outcome, the ADT (TDA) angle dataset ( Figure 10) was also significantly heterogeneous (I ^2^ = 85%, p<0.001), yet the pooled OR being insignificant. Herein, the pooled effect size indicated lower values of the ADT angle among diabetic patients (g = -0.27, 95% C.I. = -0.68–0.13, z = 1.32, p = 0.19).

Analysis of publication bias via funnel plots and related tests are depicted in Supplementary file S3, which indicate that it is unlikely that any significant publication bias occurred, but the necessity of considering several underlying factors from various angles is imperative, as discussed in the later sections. Sensitivity analysis for each dermatoglyphic variable & T2DM revealed that none of the studies influenced the results substantially.

## Discussion

The current study is supportive of T2DM being a polygenic multi-factorial disorder in which both genetic & environmental epigenetic insults cumulatively contribute to the disorder. The overall trend of reduction in digital & palmar ridge counts, considered to be directly proportional to the growth rates during the 1 ^st^ two trimesters of gestation, indicate abnormally delayed growth in diabetic cases than expected under normal conditions ^64^.

Similarly, the ATD angle is related to the axial triradius t position that migrates from the centre to the lower proximal portion of the palm during the early stages of gestation ^65^. Thus, distally deviating t positions imply prematurely halted t migrations or delayed developmental outcomes, resulting in increased ATD angles. The current analysis has observed increased (albeit insignificant) ATD increases among T2DM cases. Such findings evidence mild distortions of development during the early phases of gestational development.

### The timing of epigenetic disturbances

One advantage of DG is that each variable is formed over a given gestational timeframe, thus enabling an estimation of the relative timing of developmental insults. This entails an assumption that the stressors specifically affecting an organ or system linked to the etiology of the disorder in question also had parallel bearings on the development of DG.

Regarding the fingerprint discrepancies, the current analysis has revealed that individuals with T2DM manifested significantly reduced loop patterns, counterbalanced by elevated whorl & arch frequencies. Evidences attest that pattern determination occurs much earlier than the ridge proliferation, possibly influenced by the volar pad’s shape, the embryonic epidermal axon development, or a combination of both ^18, 66^. This asserts that patterns develop as early as weeks 6–11, thus indicating that the results of fingerprint discrepancies in this study evidence mid-to-late 1 ^st^ trimester insults on the developing fetus.

Further, for comparison purposes, the arch & whorl patterns were pooled together regarding them as a common group of the “non-loops” & analyzed, which yielded heterogeneous but significant effect sizes [OR = 1.31; 95% C.I. = 1.02–1.68, z = 2.15, p = 0.03; I ^2^ = 98%, P < 0.01], as depicted in the Supplementary file S3.

Similarly, slightly reduced digital & palmar ridge counts were found to be characteristic of T2DM. Finger ridge counts are indicative of the rate of fetal growth during ridge development which occurs from the weeks 10.5 to 17, while palmar ridge counts (TABRC) are indicative of slightly later outcomes occurring over a wider timeframe and prone to reflect effects of non-shared environmental factors ^18, 64, 65^. Thus, the insignificant findings of TFRC & TABRC reductions, coupled with the significantly reduced AFRC (though the number of studies assessing this variable is low), are indicative of disruptive events occurring during gestation roughly between weeks 11 & 20, but of lesser effects as compared to the weeks prior to this.

Unlike disorders like schizophrenia, wherein epigenetic stressors have been attributed to prevail throughout the entirety of the gestational timeframe ^29^, the results of significant fingerprint pattern deviations and the insignificant TFRC, TABRC, and palmar angle discrepancies suggest that the insults must primarily be abundant during the early stages of gestation in T2DM. More specifically, events occurring during the mid-to-late 1 ^st^ trimester have been underscored.

This stage of development witnesses key events of the endocrine pancreas development, whereby epigenetic insults could have maximum effects to result in structural & functional pancreatic defects. This period includes critical events in the prenatal development of the pancreas, including: development & fusion of ventral & dorsal primordial pancreatic buds (weeks 4–8), differentiation of the first insulin producing β-cells (weeks 7–9), as well as the clustering of endocrine cells & initiation of their subsequent association with the developing ducts of the pancreas (weeks 10–12) ^67^. Important tissues developing parallel with the pancreas (the Notochord & Mesenchyme), as well as key signaling pathways (Hedgehog & Notch) have also been shown to influence pancreatic organogenesis & underlying β-cell functioning ^67, 68^.

### Indications of an association between zygosity & fitness

Three lines of research suggest that fingerprint loops are associated with heterozygosity while arches & whorls are linked to homozygosity: (1) Population admixtures, which are known to increase genomic heterozygosity, have revealed significant loop frequency increases that are counterbalanced by non-loop (especially arch) pattern suppressions ^69, 70^; (2) The occurrence of inbreeding or endogamy, an opposite scenario to that of admixture & attributed to increased homozygosity, have been shown to decrease the loops while elevating the frequency of both non-loops ^71, 72^; (3) The classical inheritance model of fingerprint patterns attributes two of the seven genes with cumulative bearings on multiple (all) fingers, both of which are dominant, favor the non-loop outcomes in the homozygous states. Further, loops have been shown to be an attribute of heterozygous conditions for most of the remaining five genes in this model ^16^.

As advocated by the “heterozygote advantage” hypothesis ^73^, increased genomic homozygosity is associated with less viability, while an improved overall genetic fitness is attributed to increased heterozygosity. The decreased loop frequency together with the increased whorl & arch frequencies presumably represent increased homozygosity paralleled by a decreased heterozygosity in the T2DM, thus implying an overall decreased fitness in the former.

This can be interpreted via the concept of developmental homeostasis. An organism is regarded as developmentally stable based on how adequately it buffered against epigenetic disturbances during prenatal development, a capacity dependent on the interaction of genes with environmental conditions ^74^. With respect to the genotypic influences, homozygosity has been positively associated with decreased buffering capacity, while more genetically heterozygous organisms have been shown to be more resistant to developmental noise ^75^.

All in all, this zygosity-fitness-fingerprint correlation is in accordance with a string of reports on DG in T2DM & associated factors such as obesity that incorporate variables that are more potent indicators of developmental deviations associated with reduced buffering capacities, including fluctuating asymmetry, dR45 (ridge-count difference between the 4 ^th^ & 5 ^th^ fingertips), and Md15 (mean RC both thumbs minus the mean RC of both little fingers) ^22– 26^.

Supplementary evidence also originates from the previously discussed admixture changes. Heterozygosis underlying admixture was shown to be directly proportional to mean TFRC ^70^, from which it can be hypothesized that relative reductions in TFRC are indicative of increased homozygosis. The current analysis has shown that the T2DM group manifested reduced TFRC means which, though yielding non-significant pooled effect sizes, are in agreement with this hypothesis.

## Limitations

Even with the exhaustive search protocols and stringent analysis methods characteristic of the current analysis, certain limitations of the study *per se*, as well as individual studies included in the analysis need be outlined:

(1) Gender & ethnic discrepancies in DG are inherent features of normal populations ^76, 77^. Since the individual reports included consisted of samples of mixed gender and/or ethnic affiliations, there lies a possibility that such polymorphisms masked some discrepancies possessing discriminating powers between the cases & controls that would otherwise have been evident.
(2) Certain studies had underscored the presence of gender wise and/or right-left discrepancies among cases & controls for some of the dermatoglyphic variables. However, only the pooled data (for the right & left sides, as well as the males & females) was used in this analysis since the majority of the studies didn’t provide such stratified data. This poses the possibility of neglecting markers of true discriminating powers for a specific gender or side.
(3) It is known that T2DM is very heterogeneous, with distinct subtypes with parallel variations in underlying genetic determinants, such as maturity-onset diabetes of youth (MODY), latent adult-onset autoimmune diabetes, & diabetes secondary to rare genetic disorders shown to be part of the syndrome ^3, 8, 13^. Specific details, such as the ages-of-onset and other relevant specifications important in the determination of the relative homogeneity of the participants are virtually absent from all of the studies. This could be one possible explanation for the heterogeneity of the findings within a given variable type among the studies.
(4) Similarly, the co-morbidity of disorders closely associated with DM, such as hypertension and cardiac disorders, were taken into consideration by a very limited number of studies only. This occurrence has been given little coverage, even though such co-morbidity is expected to be a prevalent event ^78^, and the fact that each of these disorders are determined by distinct genetic & developmental outcomes parallel with the underlying deviations in the DG manifestations being distinct ^79^.
(5) A number of reports were not included as they were not available as free (open-access) articles. A few were also noted to bear inconsistent or incomplete data, with corresponding authors unavailable or unwilling to aid in alleviating this obstacle.

## Conclusions & recommendations

T2DM is orchestrated by the cumulative effects of a polygenic system with significant contributions from both pre- & postnatal environmental factors. Results of the current meta-analysis are indicative of epigenetic insults significantly linked to the 1 ^st^ trimester, to a lesser extent on factors of the mid-second trimester, but least likely linked to those beyond the mid-second trimester in T2DM. Further, reduced heterozygosis possibly associated with a decreased buffering capacity to epigenetic stressors (developmental noise) during prenatal development has also been noted among the diabetic group. Based on these findings, the author recommends that future research aimed at expounding the prenatal origins of T2DM, as well as developing novel therapeutic methods, should focus on the early stages of endocrine pancreatic development.

Further, the Dutch Hunger Winter Families Cohort studies ^80^ have indicated the possibility of such a link between T2DM & prenatal environment, evident in the Md15 dermatoglyphic variable, to be associated with seasonal factors. An involvement of seasonal circumstances such as vitamin D or melatonin levels, as well as fetal toxicity caused by-products of surface water chlorine treatment has been hypothesized ^26^. It is recommended to undertake parallel studies from other global areas less prone to seasonality before ascertaining such hypotheses.

Finally, the author highlights on the necessity of advancing the currently existing knowledge on the molecular genetics of DG *per se,* as this would profoundly enhance their applicability as research tools to assess the genetic epidemiology of chronic disorders.

## Supplementary materials

### Supplementary file S1.

PRISMA Checklist

### Supplementary file S2.

Studies excluded from the meta-analysis, with the respective justifications

### Supplementary file S3.

Funnel-plots for testing publication bias
