# Supplementary material for: Dermatoglyphic meta-analysis indicates early epigenetic outcomes & possible implications on genomic zygosity in type-2 diabetes
Source: F1000Res. 2015 Aug 24;4:617. [Version 1] doi: 10.12688/f1000research.6923.1 (PMC5527987; doi:10.12688/f1000research.6923.1)
Supplement: Supplementary file 2 [file f1000research-4-7455-s0001.tgz › c93263d0-a887-4511-9c55-22e9c9f5d303.docx]

**Table S1**- Studies excluded from the meta-analysis **^a^**, with the respective justifications **^b^**

| **Author(s)** | **Primary Reason for Exclusion** |
| --- | --- |
| Barta et al., 1978; Eswaraiah & Bali, 1977; Saksena et al., 1979; Vera et al., 1995; Verbov, 1973; Bets et al., 1994; Dziuba, 1973; Chakravartti, 1969; Erlick et al., 1983; Singh et al., 1988; Sant et al., 1983; Panda et al., 2004; Shubha et al., 2004; Rajib et al., 2015 | Not open-access or article not freely available (or abstracts only) |
| Platilová et al. 1996; Bodnar & Bortnichuk 1977; Segredo GJM. 1975; Vormittag et al. 1996; Yanhua et al. 1990; Khamraeva & Khamraeva (1985) | Article not available in English |
| Ravindranath et al. 2005 | Raw data or mean scores not available |
| Ziegler et al. 1993; Buti et al. 1972; Shariatzadeh et al. 2002; Nezhad et al. 2010; Shield et al. 1995; Padmini et al 2011; Ţarcă & Tuluc 2005 | Not purely T2DM diagnoses (T1DM or a combination of the two) |
| Ţarcă 2006; Shivaleela et al. 2013; Igbigbi et al. 2001 | Unique Dermatoglyphic measure, control population non-standard, or other related reason |

^a^ Some studies excluded from MA, but included in the systematic review & discussions

^b^ Some studies excluded for multiple reasons
