# Supplementary material for: Dermatoglyphic meta-analysis indicates early epigenetic outcomes & possible implications on genomic zygosity in type-2 diabetes
Source: F1000Res. 2015 Aug 24;4:617. [Version 1] doi: 10.12688/f1000research.6923.1 (PMC5527987; doi:10.12688/f1000research.6923.1)
Supplement: Supplementary file 3 [file f1000research-4-7455-s0002.tgz › ed9bae12-93b4-46a8-bca9-16b73bd7de37.docx]

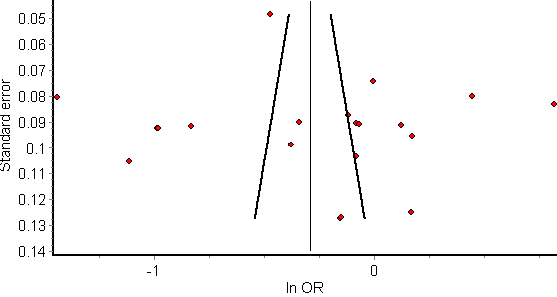


**Figure S1**- Funnel plot for publication bias in studies of the association between Loop Fingerprint Patterns & T2DM


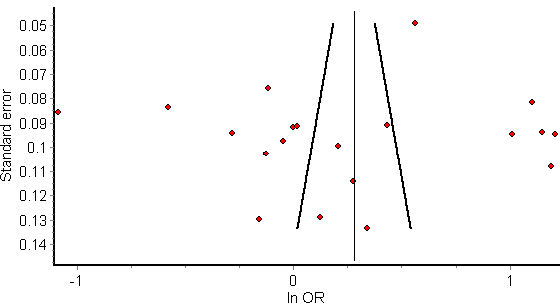


**Figure S2**- Funnel plot for publication bias in studies of the association between Whorl Fingerprint Patterns & T2DM


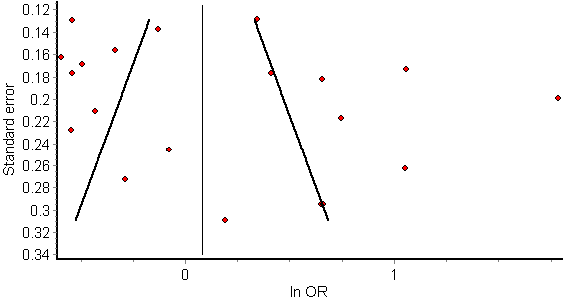


**Figure S3**- Funnel plot for publication bias in studies of the association between Arch Fingerprint Patterns & T2DM


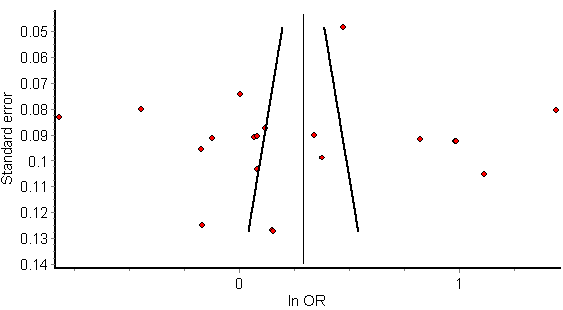


**Figure S4**- Funnel plot for publication bias in studies of the association between Non-Loop Fingerprint Patterns & T2DM


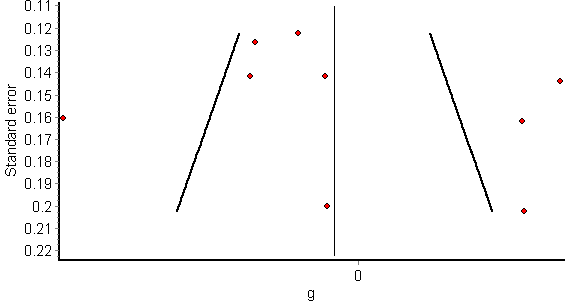


**Figure S5**- Funnel plot for publication bias in studies of the association between TFRC & T2DM


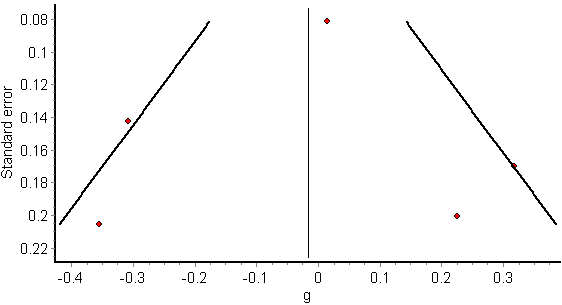


**Figure S6**- Funnel plot for publication bias in studies of the association between TABRC & T2DM


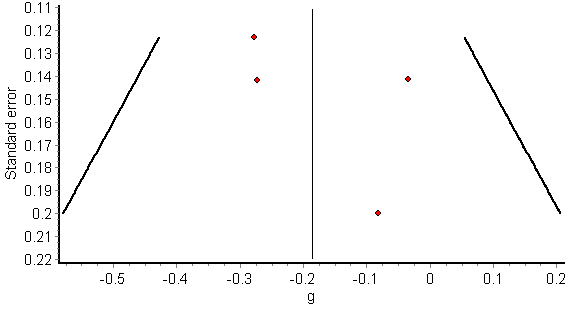


**Figure S7**- Funnel plot for publication bias in studies of the association between AFRC & T2DM


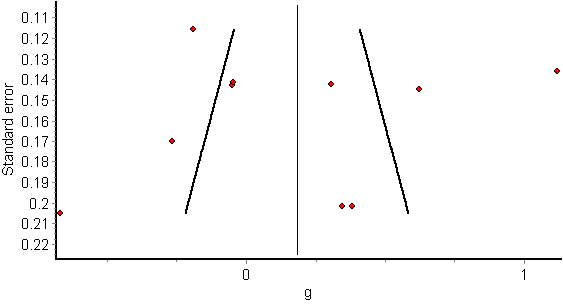


**Figure S8**- Funnel plot for publication bias in studies of the association between ATD angle & T2DM


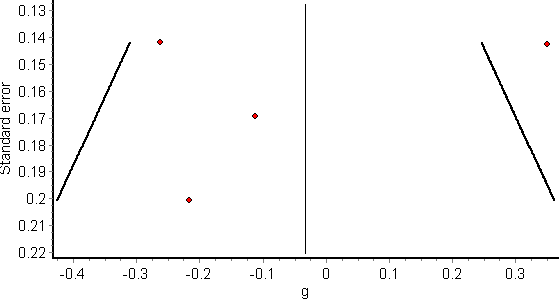


**Figure S9**- Funnel plot for publication bias in studies of the association between DAT angle & T2DM


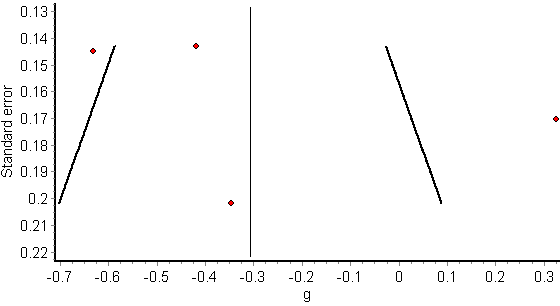


**Figure S10**- Funnel plot for publication bias in studies of the association between TDA angle & T2DM
