# Supplementary material for: Dermatoglyphic meta-analysis indicates early epigenetic outcomes & possible implications on genomic zygosity in type-2 diabetes
Source: F1000Res. 2015 Aug 24;4:617. [Version 1] doi: 10.12688/f1000research.6923.1 (PMC5527987; doi:10.12688/f1000research.6923.1)
Supplement: Peer review history

# Peer review: Referee response for version 1

**DOI:** 10.5256/f1000research.7455.r17932

**Published:** 2016-12-12

**Authors:** Tarimobo Otobo

**Affiliations:** 1 Institute of Medical Sciences, University of Toronto, Toronto, ON, Canada; 2 Developmental and Stem Cell Biology, Peter Gilgan Centre for Research and Learning, Hospital for Sick Children, Toronto, ON, Canada

The title is properly framed and reflective of the study, with a precise summary. The study design and methodology meet the standards of meta-analysis. The use of dermatoglyphics as a surrogate marker of developmental instabilities is well documented in literature. However, the correlation is equivocal. But the author has been able to present a balanced and objective report within the limitations of the analyzed reports and this study.

 The article has been reported with a reasonable depth of knowledge, providing the necessary information for replication.

I have read this submission. I believe that I have an appropriate level of expertise to confirm that it is of an acceptable scientific standard.

# Peer review: Referee response for version 1

**DOI:** 10.5256/f1000research.7455.r10489

**Published:** 2015-11-11

**Authors:** Buddhika Wijerathne

**Affiliations:** 1 Department of Forensic Medicine, Faculty of Medicine and Allied Sciences, Rajarata University of Sri Lanka, Anuradhapura, Sri Lanka

The main objective of this paper is to make a systematic review and meta-analysis of the association between dermatoglyphics and Type 2 Diabetes Mellitus. The systematic review was conducted in appropriate manner.

It is true that poor study designs, incorrect sample selections, overlooked confounding variables, made the reliability of many appraised studies questionable. The author has mentioned this in the limitation section. However, as this article is a systematic review and meta-analysis it is better to use a checklist like STROBE to assess the methodological quality of the observational studies. ( http://www.strobe-statement.org/).

In results section it mentioned “Of the 27 studies, respectively 20, 9, 4, 5, 10, 4 and 4 studies reported valid & usable data on the”

What are these numbers? Are they references?

ATD angle is not a good variable in Dermatoglyphics studies for various reasons and therefore needs a little extension of discussion. In a recent review on hypertension and dermatoglyphics we have discussed this issue in detail. DOI: 10.1186/s40101-015-0065-3.

I have read this submission. I believe that I have an appropriate level of expertise to confirm that it is of an acceptable scientific standard.
